# Supplementary material for: Phylogenetic Analysis to Explore the Association Between Anti-NMDA Receptor Encephalitis and Tumors Based on microRNA Biomarkers
Source: Biomolecules. 2019 Oct 5;9(10):572. doi: 10.3390/biom9100572 (PMC6843259; doi:10.3390/biom9100572)
Supplement: Supplementary file 1 [file biomolecules-09-00572-s001.pdf]

MATLAB code of plotting a phylogenetic tree of 3 miRNAs

```
fastawrite('m129','miR-129','GGAUCUUUUUGCGGUCUGGGCUUGCUGUUCCU
CUCAACAGUAGUCAGGAAGCCCUUACCCCAAAAAGUAUCU');
R129=fastaread('m129');

fastawrite('m555','miR-555','GGAGUGAACUCAGAUGUGGAGCACUACCUUUG
UGAGCAGUGUGACCCAAGGCCUGUGGACAGGGUAAGCUGAACCUCUGA
UAAAACUCUGAUCUAU');
R555=fastaread('m555');

fastawrite('m19a','miR-19a','GCAGUCCUCUGUUAGUUUUGCAUAGUUGCACU
ACAAGAAGAAUGUAGUUGUGCAAAUCUAUGCAAAACUGAUGGUGGCCU
GC');
R19a=fastaread('m19a');

seqs = [R129, R555, R19a];
distances = seqpdist(seqs,'method','jukes-cantor','Alphabet', 'NT');
%distances = seqpdist(seqs,'method','alignment-score','Alphabet', 'NT');
%distances = seqpdist(seqs,'method','p-distance','Alphabet', 'NT');
phylotree = seqlinkage(distances,'average',seqs);
%phylotree = seqlinkage(distances,'single',seqs);
%phylotree = seqlinkage(distances,'median',seqs);
view(phylotree)
```

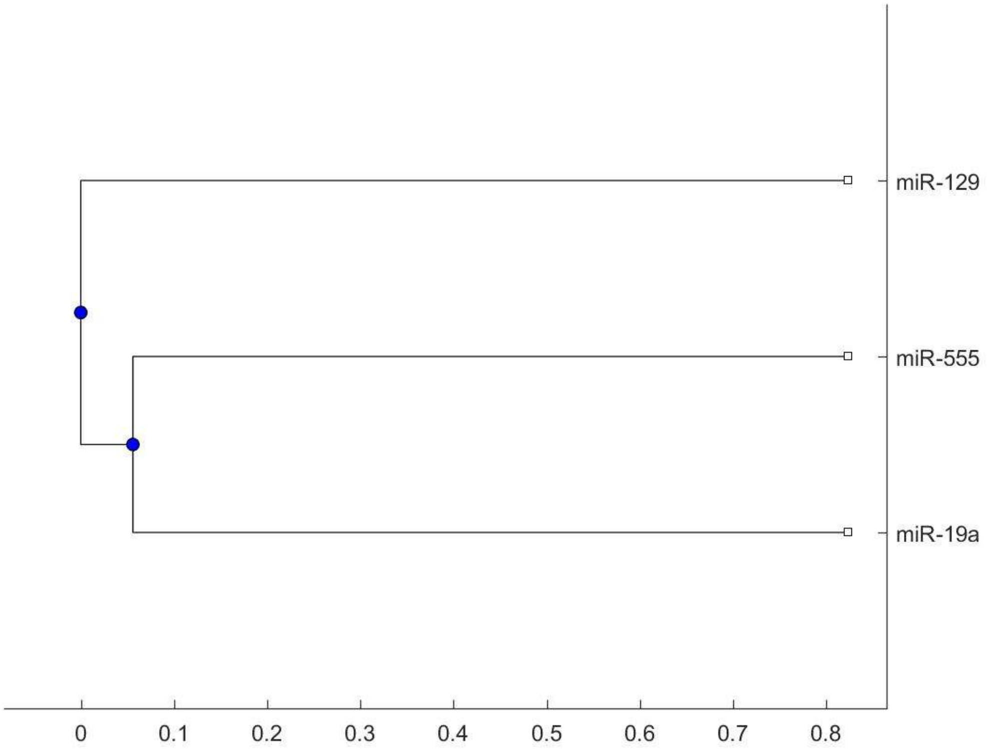
miR-129
miR-555
miR-19a
0
0.1
0.2
0.3
0.4
0.5
0.6
0.7
0.8
